# Error awareness as evidence accumulation: effects of speed-accuracy trade-off on error signaling

**DOI:** 10.3389/fnhum.2012.00240

**Published:** 2012-08-13

**Authors:** Marco Steinhauser, Nick Yeung

**Affiliations:** ^1^Department of Psychology, Catholic University of Eichstätt-IngolstadtEichstätt, Germany; ^2^Department of Psychology, University of KonstanzKonstanz, Germany; ^3^Department of Experimental Psychology, University of OxfordOxford, UK

**Keywords:** error awareness, performance monitoring, event-related potentials, single-trial analysis, error-related negativity, error positivity

## Abstract

Errors in choice tasks have been shown to elicit a cascade of characteristic components in the human event-related potential (ERPs)—the error-related negativity (Ne/ERN) and the error positivity (Pe). Despite the large number of studies concerned with these components, it is still unclear how they relate to error awareness as measured by overt error signaling responses. In the present study, we considered error awareness as a decision process in which evidence for an error is accumulated until a decision criterion is reached, and hypothesized that the Pe is a correlate of the accumulated decision evidence. To test the prediction that the amplitude of the Pe varies as a function of the strength and latency of the accumulated evidence for an error, we manipulated the speed-accuracy trade-off (SAT) in a brightness discrimination task while participants signaled the occurrence of errors. Based on a previous modeling study, we predicted that lower speed pressure should be associated with weaker evidence for an error and, thus, with smaller Pe amplitudes. As predicted, average Pe amplitude was decreased and error signaling was impaired in a low speed pressure condition compared to a high speed pressure condition. In further analyses, we derived single-trial Pe amplitudes using a logistic regression approach. Single-trial amplitudes robustly predicted the occurrence of signaling responses on a trial-by-trial basis. These results confirm the predictions of the evidence accumulation account, supporting the notion that the Pe reflects accumulated evidence for an error and that this evidence drives the emergence of error awareness.

Continuous monitoring of action outcomes is crucial for achieving optimal performance. Evidence for a performance monitoring system involved in error detection has been provided by studies examining event-related potentials (ERPs). In these studies, errors in simple choice tasks have been shown to elicit a negative deflection at fronto-central electrodes called the error negativity (Ne, Falkenstein et al., 1990) or error-related negativity (ERN, Gehring et al., 1993), that is followed by a positive deflection at posterior electrodes called the error positivity (Pe, Falkenstein et al., 1990). Whereas early theories suggested that the Ne/ERN directly reflects error detection (Falkenstein et al., 1990; Gehring et al., 1993), it has recently been proposed that the Ne/ERN is related to other aspects of error processing like response conflict (Yeung et al., 2004) or reinforcement learning (Holroyd and Coles, 2002). In contrast, the Pe has been suggested to be a correlate of conscious error processing or error awareness (e.g., Falkenstein et al., 2000). The goal of the present study was to contribute to a deeper understanding of how error awareness is achieved by investigating the relationship between the Pe and behavioral measures of error awareness.

In recent years, the neural correlates of error awareness have been investigated in a number of studies (for an overview, see Ullsperger et al., 2010). A frequently-used method for measuring error awareness is the so-called error signaling paradigm, initially introduced by Rabbitt and colleagues (Rabbitt, 1968, 2002). In this paradigm, participants perform a speeded choice task (the primary task). After each response, they have to press a signaling key whenever they think that they have made an error. Using this paradigm, several studies have investigated the relation between signaling responses and the amplitude of error-related ERP components. Whereas the majority of studies have reported an increased Pe for signaled errors relative to unsignaled errors (Nieuwenhuis et al., 2001; Endrass et al., 2005, 2007; Overbeek et al., 2005; O'Connell et al., 2007; Shalgi et al., 2009; Steinhauser and Yeung, 2010; Dhar et al., 2011; Hewig et al., 2011; Hughes and Yeung, 2011; Wessel et al., 2011; Murphy et al., 2012), only a few studies found such a result for the Ne/ERN (e.g., Maier et al., 2008; Steinhauser and Yeung, 2010; Wessel et al., 2011).

## Error awareness as evidence accumulation

Although these findings suggest a relationship between the Pe and error awareness, they are less informative regarding the specific role of the Pe in the emergence of awareness. To address this question, we recently proposed that error awareness can be conceptualized as a decision process, in which the available evidence that an error has occurred is accumulated until a decision criterion is reached (Steinhauser and Yeung, 2010). Within this framework, we asked whether error-related brain activity reflects the accumulated evidence that an error has occurred, or the output of this decision. By varying the decision criterion of error signaling, we were able to test specific predictions associated with each hypothesis. We found that although a higher decision criterion led to fewer signaled errors, it was not associated with a reduced Pe amplitude. This finding implies that average Pe amplitude does not reflect the number of signaled errors, and thus, the output of the decision process. We further found that a higher decision criterion was associated with a larger Pe amplitude if signaled errors were considered. This result reflects the fact that with a high criterion, more evidence for an error is required to exceed this criterion, which is consistent with the assumption that the Pe reflects the accumulated evidence that an error has occurred. Further support for this conclusion was provided by single-trial analyses. Using a logistic regression approach (Parra et al., 2002, 2005), we derived a single-trial measure of the Pe amplitude. As predicted by our evidence accumulation account, this “error signal” could be used to robustly predict whether or not an error would be followed by an error signaling response. Taken together, these results suggest that the Pe does not reflect whether an error was consciously detected or not but rather reflects the accumulation of evidence for an error that precedes the emergence of awareness. Whether a given amount of evidence (i.e., a given Pe amplitude) on a trial leads to error awareness depends on the decision criterion.

## Computational accounts of error detection

Whereas our previous study provides a framework for explaining the relation between the Pe and error awareness, it did not specify the process that delivers the internal evidence for an error, nor did it make assumptions about the nature of this evidence. Potential answers to these questions have been provided by theories of error detection in decision-making (for an overview, see Yeung and Summerfield, 2012). In recent years, two accounts have been proposed which themselves are based on evidence accumulation models: the response monitoring account (Steinhauser et al., 2008; see also Rabbitt and Vyas, 1981) and the conflict monitoring account (Yeung et al., 2004). These accounts share the assumption that response selection in choice tasks occurs when evidence for a response exceeds a response criterion. A crucial feature of evidence accumulation models is their strong self-correction tendency. After an error has occurred due to noise in the accumulation process, continued evaluation of the stimulus usually ensures that accumulated evidence for the correct response eventually exceeds that for the incorrect response. The two accounts mainly differ with respect to which aspect of self correction provides the diagnostic feature that underlies error detection:

- The response monitoring account (Steinhauser et al., 2008) assumes that performance monitoring registers that a second response (i.e., an internal correction response) exceeds the primary task's response criterion. However, when Steinhauser et al. (2008) fitted a model of this account to empirical data, it turned out that only about 60% of trials with an internal correction response also led to a signaling response. This suggests that an internal correction response does not directly trigger error awareness (which implies that the response criterion of the primary task does not correspond to the decision criterion associated with error awareness). It rather provides the internal evidence for an error, which forms the basis of the error decision, and which could lead to error awareness or not^1^.
- The conflict monitoring account (Yeung et al., 2004) assumes that performance monitoring registers response conflict which occurs when strong evidence is accumulated for multiple responses—a condition that necessarily accompanies self correction. This response conflict is accumulated until it reaches another criterion, which then leads to error awareness. Accordingly, this account assumes that response conflict rather than an internal correction response provides the internal evidence for an error.

In a simulation study, Steinhauser et al. (2008) investigated whether these two accounts can predict the latencies and frequencies of error signaling responses in an experiment in which the speed-accuracy trade-off (SAT) of the primary task was manipulated. To derive predictions, response monitoring and conflict monitoring were implemented in a connectionist model. Following standard theories of SAT (for an overview, see Bogacz et al., 2010), the effects of speed pressure were simulated by varying the primary task's response criterion. For such a case, one might expect that slower responding is beneficial for performance monitoring, for instance, because it leads to a better representation of the correct response (e.g., Falkenstein et al., 2000). In contrast to this intuition, the simulations revealed that both accounts predict the opposite: with an increased response criterion and, thus, slow responding, both accounts predicted that fewer errors were signaled and that the latency of error signaling was increased. The analysis of simulation data revealed that, for both accounts, this pattern was due to the fact that evidence for an error was weaker: response monitoring predicted that an increased primary task's response criterion reduces the probability and prolongs the time until an internal correction response exceeded this criterion. Similarly, conflict monitoring predicted that an increased primary task's response criterion reduces and delays response conflict after an error. The latter result obtains because a larger response criterion implies that, at the time of the error response, there is a larger difference between the accumulated evidence for the incorrect response alternative and that for the correct response alternative. This impairs the emergence of response conflict after the error, because with this larger initial difference, the self-correction tendency of the primary task's response selection process requires more time until enough evidence is accumulated for the correct response to cause a response conflict with the already accumulated evidence for the incorrect response.

The experimental data by Steinhauser et al. (2008) confirmed these predictions by showing that low speed pressure, and thus a high response criterion, led to fewer signaled errors and delayed signaling responses (for a similar result, see Shalgi et al., 2007). Because the quantitative fit of the response monitoring model was much better than that of the conflict monitoring model, it was concluded that, at least in this experiment, error signaling was driven by response monitoring. Most importantly for the present study, however, this finding demonstrates that response monitoring and conflict monitoring not only provide specific assumptions about the nature of the internal evidence for an error, they also make specific predictions how this evidence is influenced by experimental variables like SAT.

## The present study

In the present study, we used the model predictions of Steinhauser et al. (2008) to test a crucial prediction of our evidence accumulation account of error awareness. Whereas our previous study (Steinhauser and Yeung, 2010) manipulated the decision process itself, we now manipulated the evidence feeding into this decision, and asked whether the amplitude of the Pe varies as a function of the strength and latency of the evidence. To achieve this, we manipulated the SAT of a primary task and investigated its influence on error signaling and the Pe. Following the simulation results of Steinhauser et al. (2008), we predicted that low speed pressure should be associated with weaker evidence for an error. As a consequence, if the evidence accumulation account is valid and the Pe reflects the evidence for an error, then low speed pressure should also imply a reduced Pe amplitude^2^.

Interestingly, previous studies investigating the effects of SAT on error-related brain activity have typically found the opposite result: Ne/ERN and Pe amplitudes in these studies were increased when accuracy was prioritized over speed (e.g., Gehring et al., 1993; Arbel and Donchin, 2009). However, these studies used paradigms in which SAT shifts were associated with changes in selective attention (for a discussion, see Yeung et al., 2004), and in error significance. In the present study, we manipulated the SAT in a brightness discrimination task in which no selective attention was necessary because no distractor stimuli were used (Steinhauser and Yeung, 2010). Moreover, SAT was manipulated by means of a speed pressure instruction without emphasizing accuracy, and thus, without affecting the subjective significance of an error.

Speed pressure was varied across two conditions, a low speed pressure (lowSP) condition and a high speed pressure (highSP) condition. According to the model predictions of Steinhauser et al. (2008), lower speed pressure should result in weaker evidence for the occurrence of an error. As discussed above, this change is not a direct consequence of the reduced response speed, but rather reflects the increased response criterion in the primary task. If evidence is weaker in the lowSP condition, signaling responses should be less frequent, and the latency of these signaling responses should be increased. Moreover, decreased accumulated evidence in the lowSP condition should be reflected in a smaller Pe. Similar to Steinhauser and Yeung (2010), we tested these predictions for the Pe for all error trials as well as for signaled error trials only. If we found similar effects for all errors and for signaled errors, this would show that changes in Pe amplitudes are actually due to changes in the strength of accumulated evidence rather than changes in the number of signaled errors.

## Method

### Participants

Eighteen right-handed participants (12 female) between 19 and 24 years of age (mean 21.1) with normal or corrected-to-normal vision participated in the study. Participants were recruited at the University of Konstanz for course credit or a payment of 6 Euro per hour, and were paid an additional performance-dependent bonus.

### Task and procedure

We used the paradigm introduced by Steinhauser and Yeung (2010), in which participants first performed a brightness discrimination task and then were prompted to make a signaling response when they thought they had made an error. All stimuli were presented on a screen with a resolution of 1080 by 1024 pixels and at a viewing distance of 60 cm. The stimuli in the primary task consisted of two boxes presented on a black background above and below a white fixation cross. Each box consisted of a 64-by-64 array of randomly arranged white and black pixels, with new arrays generated on each trial. Discrimination difficulty depended on the relative proportions of white and black pixels in the two boxes. In contrast to Steinhauser and Yeung (2010), the difficulty level was set to a constant value throughout the experiment, with 55% white pixels in the brighter box compared with 45% in the darker box.

**Figure 1:**
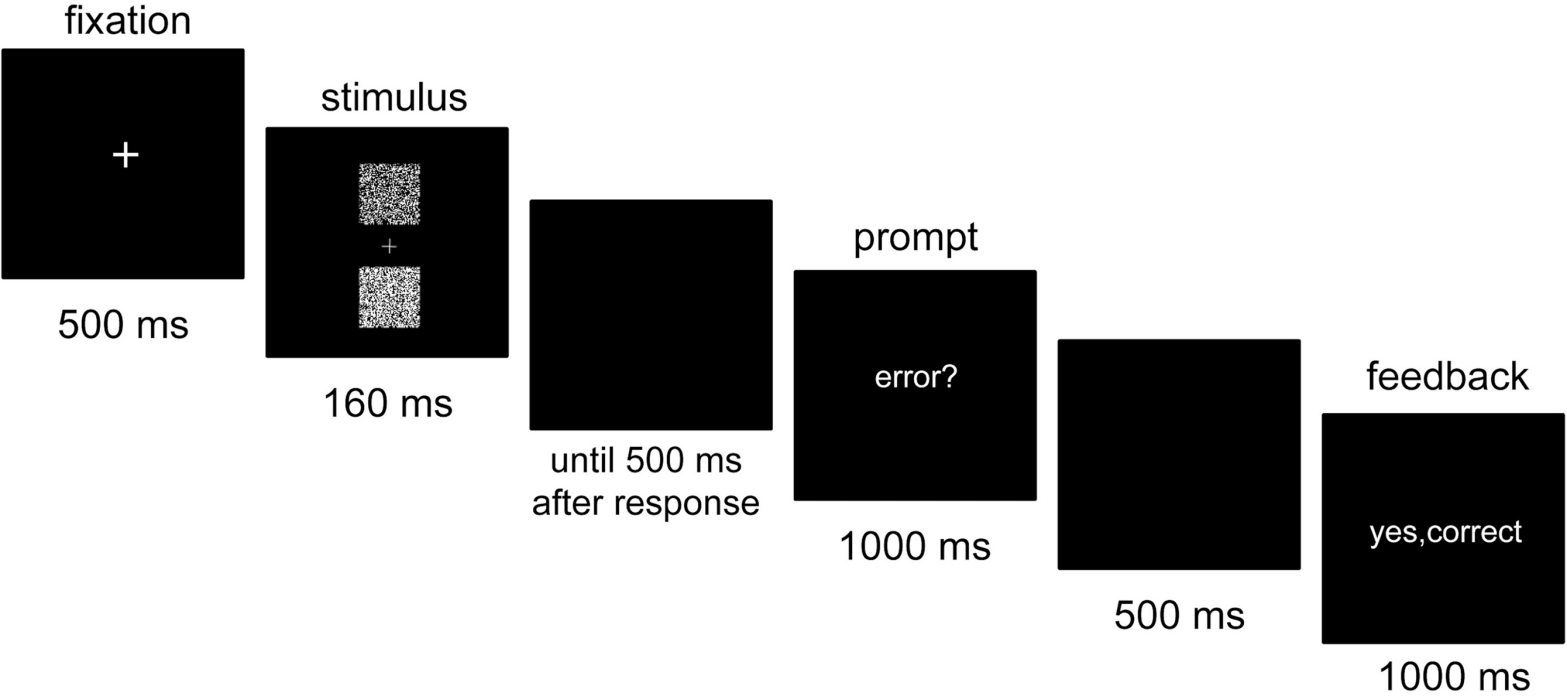
**Sequence of stimulus events in a trial**. Participants were first required to indicate which of two boxes in the stimulus was brighter. Following the error prompt, they pressed a signaling key if they judged that their primary task response was an error.

Figure 1 depicts a sample trial. First, a white fixation cross was centrally presented for 500 ms. Then, the stimulus of the primary task appeared for 160 ms, followed by a blank screen. The primary task response was provided by pressing one of two keys on a standard keyboard: the “T” key with the left index finger when the upper box was brighter and the “G” key with the right index finger when the lower box was brighter. 500 ms after the response, the word “error?” was centrally presented for 1000 ms. During that time, participants were instructed to press the space bar with their right thumb if they thought that they had committed an error in the primary task. Then another blank screen appeared for 500 ms, followed by a feedback screen presented for 1000 ms.

The feedback screen indicated the accuracy of both the primary task response and the error signaling response. If the primary task response was correct, and was not followed by an error signaling response, the feedback indicated “yes, correct” in green (correct rejection). If the primary task response was correct, but was followed by an erroneous error signal, the feedback indicated “no, correct” in red (false alarm). If an incorrect primary task response was followed by an error signaling response, the feedback indicated “yes, error” in green (hit). Finally, if an incorrect primary task response remained unsignaled, the feedback indicated “no, error” in red (miss). In experimental blocks, the feedback screen additionally indicated the amount of win or loss (e.g., “+2” or “−2”) in this trial.

The experiment consisted of three parts: a practice part, a low speed pressure part, and a high speed pressure part. The practice part consisted of five blocks: first, three blocks of 30 trials were conducted in which only the primary task was practiced and no feedback was provided after each trial. Participants were instructed to respond as quickly and as accurately as possible. After each block (in this and the following parts), feedback about mean RT and error rate was provided. If error rate in these blocks fell below 20%, participants were instructed to increase response speed. Then, two further practice blocks were conducted in which the error signaling procedure was introduced. In these blocks, trial feedback as described above (but without indicating wins and losses) was presented. If the error rate in the final practice block fell below 20%, another practice block was conducted and participants were instructed to increase response speed. This was repeated until the required error rate was achieved. Note that although we applied an accuracy criterion in this part, only response times but never error rates were mentioned during instructions in this and the following parts. We did this to ensure that instructions did not influence the subjective significance of errors.

After the practice part, half of the participants continued with the low speed pressure part and then with the high speed pressure part. This order was reversed in the other half of the participants. Low and high speed pressure was induced only by means of instruction. Participants were instructed not to exceed an individually determined criterion RT. If the mean RT during a block was larger than this criterion RT, participants were instructed to increase response speed. In the low speed pressure blocks, criterion RT was the mean RT from the last practice block plus 50 ms. In the high speed pressure block, criterion RT was the mean RT from the last practice block minus 50 ms. Each part started with two practice blocks of 30 trials, in which participants could adapt to the instructed speed pressure. These practice blocks were followed by four experimental blocks of 60 trials each, resulting in 240 experimental trials in each speed pressure condition. In experimental trials, participants earned money for correct error signaling. They won 2 points each time they signaled on error trials (hits) or withheld from signaling on correct trials (correct rejections). They lost 2 points each time they signaled on correct trials (false alarms) or failed to signal after errors (misses). At the end of the experiment, points were converted into a monetary reward (1 point = 1 Eurocent). In the present study, this reward scheme served no specific purpose beyond encouraging accurate error signaling, but this feature makes the design comparable to our previous study (Steinhauser and Yeung, 2010).

### Data acquisition

The electroencephalogram (EEG) was recorded using a BIOSEMI Active-Two system (BioSemi, Amsterdam, The Netherlands) with 64 Ag-AgCl electrodes from channels Fp1, AF7, AF3, F1, F3, F5, F7, FT7, FC5, FC3, FC1, C1, C3, C5, T7, TP7, CP5, CP3, CP1, P1, P3, P5, P7, P9, PO7, PO3, O1, Iz, Oz, POz, Pz, CPz, Fpz, Fp2, AF8, AF4, AFz, Fz, F2, F4, F6, F8, FT8, FC6, FC4, FC2, FCz, Cz, C2, C4, C6, T8, TP8, CP6, CP4, CP2, P2, P4, P6, P8, P10, PO8, PO4, O2 as well as the left and right mastoid. The Common Mode Sense (CMS) and Driven Right Leg (DRL) electrodes were used as reference and ground electrodes. Vertical and horizontal electrooculogram (EOG) was recorded from electrodes above and below the right eye and on the outer canthi of both eyes. All electrodes were off-line re-referenced to averaged mastoids. EEG and EOG data were continuously recorded at a sampling rate of 1024 Hz, and were re-sampled to 512 Hz offline.

### Data analysis

For analysis of primary task performance and ERP data, trials from each condition were categorized as correct responses and errors. For analysis of error signaling performance, trials from each condition were categorized as correct rejections, false alarms, misses, and hits. The absolute frequencies were used to calculate the hit rate, *H* (= proportion of hits among all errors), and the false alarm rate, *FA* (= proportion of false alarms among all correct trials), for both conditions. We then estimated two parameters from Signal Detection Theory (Green and Swets, 1966; Macmillan and Creelman, 1991): the detection criterion *c*, and the sensitivity *d*'. Signaling latencies were calculated as the difference between the primary task response and the signaling response. In this way, occasional signaling responses that occurred prior to the signaling prompt were assigned a positive latency^3^.

EEG data were analyzed using EEGLAB v6.01 (Delorme and Makeig, 2004) and custom routines written in MatLab 7.0.4 (The Mathworks, Natick, MA). The data were band-pass filtered excluding activity below 1 Hz and above 30 Hz (waveforms in figures were additionally filtered with a 15 Hz low-pass filter). Epochs were extracted ranging from 500 ms before and 1000 ms after the response. Artifacts were removed using standard routines implemented in EEGLAB v6.01: first, large artifacts were identified by computing the joint probability of each epoch and excluding epochs that deviated more than five standard deviations from the distribution mean. Second, ocular artifacts were corrected by an eye movement correction procedure (Automatic Artifact Removal Version 1.3, http://kasku.org/aar/) based on a linear regression approach (Gratton et al., 1983). Baseline activity was removed by subtracting the average voltage in an interval between 400 ms and 100 ms before the response. This baseline was chosen because it precedes the onset of the Ne/ERN.

After artifact removal, the resulting waveforms included an average of 153 correct trials (range: 113–204) and 75 error trials (range: 26–113) in the highSP condition and 176 correct trials (range: 119–225) and 48 error trials (range: 9–111)^4^ in the lowSP condition. If only signaled errors were considered, there were an average of 139 correct trials (range: 89–202) and 62 error trials (range: 21–93) in the highSP condition and 163 correct trials (range: 106–224) and 37 error trials (range: 8–94) in the lowSP condition. Error-related brain activity was quantified by computing the mean amplitude of the waveform for errors in a time interval that captures the main portion of the component of interest. For the Ne/ERN, an interval from –50 to 50 ms relative to the response was used. For the Pe, an interval from 150 to 400 ms after the response was used. All components were quantified for each channel. However, statistical analysis was applied only to data from channel FCz for the Ne/ERN (for which the Ne/ERN is typically largest) and from channel POz for the Pe. The latter was chosen because the error signal found by Steinhauser and Yeung (2010) was maximal at this channel, a finding that was replicated in the present study.

Because we found that ERP differences between conditions partially reflected RT differences (due to differential influence of stimulus-locked components on response-locked data), analyses were also applied to a subset of RT matched trials. To achieve RT matching, we first identified the condition with the fewest trials (i.e., errors/lowSP) and then matched all other conditions (errors/highSP, corrects/lowSP, corrects/highSP) to this condition using the following algorithm: First, a trial from the error/lowSP condition was randomly drawn (without replacement). Second, from each other condition, the trial providing the closest match to the RT of the drawn trial was selected (without replacement) and assigned to the RT-matched sample. These steps were repeated until all trials from the error/lowSP condition were drawn. Note that only artifact-free trials were included. Therefore, mean RT of the error/lowSP condition deviates slightly from the value obtained in the initial analysis of behavioral data.

In addition, we aimed to replicate the findings by Steinhauser and Yeung (2010) that Pe amplitude predicts error signaling on a trial-by-trial level. To achieve this, a single-trial analysis was conducted using the linear integration method introduced by Parra et al. (2002) to measure error-related EEG activity with improved signal-to-noise ratio. The rationale of this method is to extract a specific spatial component of the ERP by constructing a classifier that maximally discriminates between two conditions differing in this component. Specifically, with *x(t)* being the vector of electrode activity at time *t*, we used logistic regression to compute a spatial weighting coefficient *v* such that the component

$$y(t)=v^{T}x(t)$$

is maximally discriminating between two different conditions. In the present case, we used this method to discriminate between error and correct-response trials in order to estimate error-related EEG activity on individual trials (independent of speed pressure condition). As input, we used *T* samples from each of the *N* baseline-corrected ERP epochs, resulting in a training set of size *NT*. After finding the optimal *v*, we estimated the error signal, ${\bar{y}}_{k}$, on each trial *k* by averaging across the *T* samples from each trial. This value ranges between 0 and 1, with higher values indicating a higher probability that the trial was an error.

To visualize the spatial distribution of weights of the discriminating component, we computed the coupling coefficient vector

$$a=\frac{Xy}{y^{T}y},$$

with time *t* being a dimension of the matrix *X* and the vector *y*. Coupling coefficients represent the activity at each electrode site that correlates with the discriminating component, and thus can be thought of as the “sensor projection” of that component (Parra et al., 2002, 2005).

The analysis was applied to the same time range (250–350 ms after the response) as in Steinhauser and Yeung (2010). First, classifier sensitivity was quantified in terms of *Az*-score, which corresponds to the area under the Receiver Operating Characteristic curve, with 0.5 indicating chance-level classification and 1 indicating perfect discrimination. *Az*-scores were computed for each window using split-half cross-validation, i.e., the classifier was trained on half of the trials and was then used to predict the category (correct or error) on the remaining trials. This procedure was repeated for each half of 10 random splits, and the average of these 20 values was taken as the overall sensitivity for a specific window and participant. To test whether sensitivity significantly exceeded chance level, a permutation test was applied (e.g., Philiastides et al., 2010; Steinhauser and Yeung, 2010). For each participant, a test distribution under the Null hypothesis was generated by recomputing *Az*-scores with random assignment of the correct/error categories. This procedure was repeated 100 times for each of the 20 subsets of trials from which each *Az-score* was computed. The resulting 2000 values represented the test distribution, and were used to determine critical *Az*-values associated with significance levels of 0.05 and 0.01. Overall critical *Az*-values were computed by averaging across participants.

Following Steinhauser and Yeung (2010), we used the error signal y_k_ as a neural correlate of the accumulated evidence that an error has occurred, and investigated whether this error signal can be used to predict error signaling on a trial-by-trial basis. To this end, we first calculated the mean error signal separately for each trial by averaging across values from the 20 split-half samples. Prediction of the occurrence of a signaling response was achieved using a logit regression analysis with a binary dependent variable (signaled error vs. unsignaled error) and a continuous independent variable (mean error signal). The resulting beta values were analyzed using *t*-tests and repeated measurement ANOVAs.

## Results

### Behavioral data

Behavioral data are presented in Table 1. We first analyzed primary task performance to examine whether our manipulation of SAT was successful. As expected, the lowSP condition was associated with decreased error rates, *F*_(1, 17)_ = 21.0, *p* < 0.001, increased correct RTs, *F*_(1, 17)_ = 14.0, *p* < 0.01, and increased error RTs, *F*_(1, 17)_ = 8.75, *p* < 0.01, indicating that the speed pressure manipulation led to a shift in SAT.

**Table 1:** **Behavioral performance**.

	**HighSP**		**LowSP**			
	**Mean**	**SE**	**Mean**	**SE**	***F*_(1, 17)_**	***p***
**PRIMARY TASK PERFORMANCE**												
Error rate (%)	33.1	0.03	21.5	0.03	21.0	<0.001
RT correct (ms)	338	23	412	22	14.0	<0.01
RT error (ms)	320	25	410	43	8.75	<0.01
**ERROR SIGNALING PERFORMANCE**												
Latency (ms)	703	33	756	29	5.07	<0.05
Hit rate (%)	81.7	3.6	76.6	0.05	3.71	0.07
False alarm rate (%)	1.8	0.3	1.5	0.03	0.79	0.39
Criterion c	0.56	0.09	0.73	0.11	4.53	<0.05
Sensitivity d'	3.23	0.14	3.15	0.19	0.78	0.39

Primary task error rates and response times (RTs), error signaling rates and latency, and estimated signal detection parameters for the two speed pressure conditions.

Note: SE, standard error of the mean; lowSP, low speed pressure; highSP, high speed pressure.

As predicted, this SAT shift in the primary task also influenced error signaling. Signaling RT was increased in the lowSP condition, *F*_(1, 17)_ = 5.07, *p* < 0.05. Moreover, the frequency of signaling responses was numerically reduced. Although this effect was not significant for the false alarm rates and was only marginally significant for the hit rates, *F*_(1, 17)_ = 3.71, *p* = 0.07, the estimated detection criterion—a measure that combines the two rates—was significantly increased in the lowSP condition^5^, *F*_(1, 17)_ = 4.53, *p* < 0.05. In contrast, detection sensitivity *d*' did not differ reliably across the two conditions (*F* < 1).

### Event-related potentials

The behavioral data followed a similar pattern to the one obtained in Steinhauser et al. (2008): low speed pressure for the primary task led to longer signaling RTs and a lower frequency of signaling responses. In a next step, we examined whether these behavioral effects were reflected in specific changes in the Pe and the Ne/ERN. Based on the simulations of Steinhauser et al. (2008), we predicted that weaker evidence for an error would be evident with low speed pressure. Provided that the accumulated evidence for an error is reflected by the Pe amplitude, we should therefore observe a reduced Pe amplitude in the lowSP condition. Moreover, this effect should be obtained across all error trials as well as for signaled error trials specifically. If such an effect were obtained only if all error trials were included, it could simply reflect the decreased rate of signaled errors in the lowSP condition.

**Figure 2:**
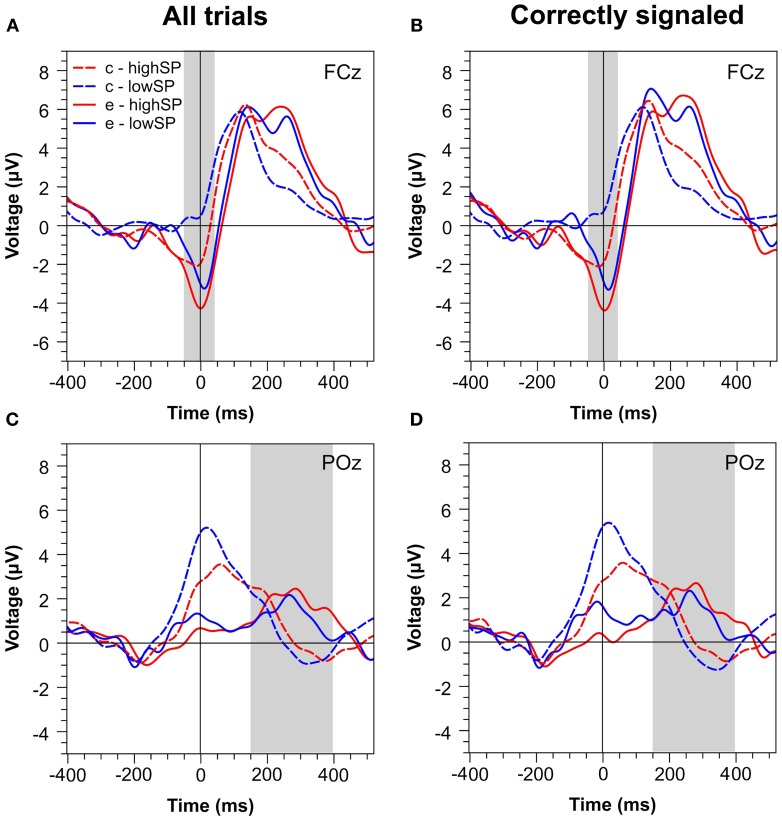
**Response-locked ERPs separately for the highSP and lowSP conditions**. **(A,C)** Mean ERP waveforms at electrodes FCz and POz for all errors and correct responses. **(B,D)** Mean ERP waveforms at electrodes FCz and POz for signaled errors and unsignaled correct responses. Zero indicates the time of the response. HighSP = high speed pressure. LowSP = low speed pressure.

Figure 2 presents waveforms at two characteristic channels, FCz and POz, for all trials (Figures 2A,C) and for trials that were correctly signaled (i.e., signaled errors and unsignaled correct trials, Figures 2B,D). The waveforms reveal strong differences between speed pressure conditions. At least for correct trials, however, these differences seem to reflect RT differences between these conditions: waveforms for correct trials in the highSP condition are delayed relative to those in the lowSP condition. This might reflect that, due to shorter RTs in the highSP condition, stimulus-locked components occur later relative to the response in this condition (Coles et al., 2001; Maier et al., 2010)—a conclusion receiving support from the observation that this effect disappeared when RT matched data were analysed (see below). At first glance, such an effect does not seem to be responsible for differences between the waveforms for error trials, at least in the time range of the Pe. However, to prevent bias of our analysis by the differential contribution of RT effects to correct and error trials, we directly compared error trials between our conditions.

**Figure 3:**
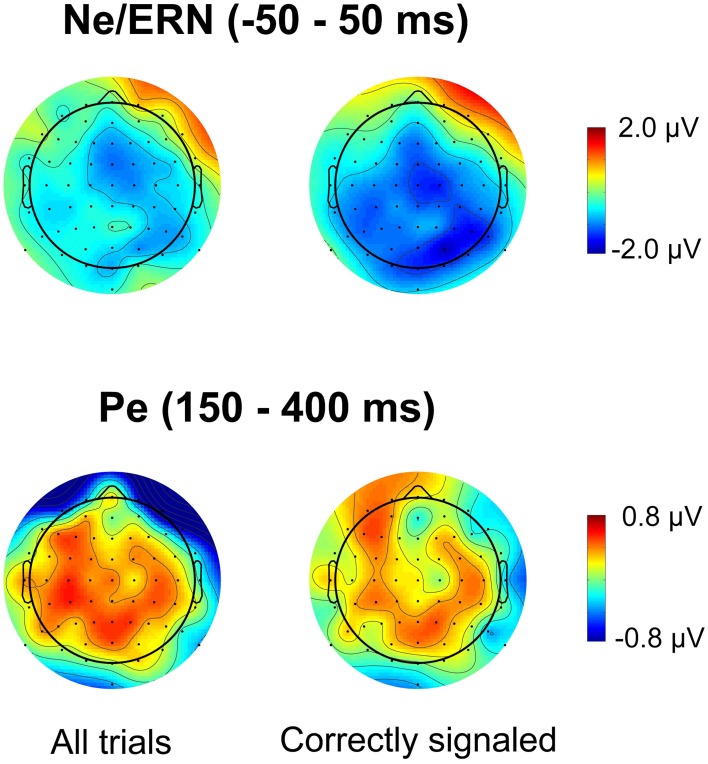
**Spatial distribution of ERPs for the difference between errors in the lowSP condition and errors in the highSP condition**. *Upper row:* Time period of the Ne/ERN (−50 – 50 ms). *Lower row:* Time period of the Pe (150–400 ms). *Left column:* Data from all errors. *Right column:* Data from signaled errors. HighSP = high speed pressure. LowSP = low speed pressure.

Figure 3 plots the spatial distribution of the difference wave between the lowSP and the highSP condition for error trials in the time range of the Ne/ERN and the Pe. For the Pe, the data reveal differences with a broad central spatial distribution, irrespective of whether all trials or only correctly signaled trials were considered. As predicted, Pe amplitude was decreased for the lowSP condition relative to the highSP condition, and this difference was significant for all trials (1.25 μV vs. 1.81 μV), *F*_(1, 17)_ = 7.57, *p* < 0.05, as well as for correctly signaled trials (1.27 μV vs. 1.83 μV), *F*_(1, 17)_ = 5.45, *p* < 0.05, at channel POz. For the Ne/ERN, we obtained a difference in the same direction at channel FCz which was marginally significant for all trials (–2.19 μV vs. –3.29 μV), *F*_(1, 17)_ = 3.19, *p* < 0.10, as well as for correctly signaled trials (–2.15 μV vs. –3.41 μV), *F*_(1, 17)_ = 3.40, *p* < 0.10.

As already mentioned, differences between waveforms in our speed pressure conditions partially reflect RT differences. Although this seems to hold mainly for correct trials, we cannot exclude the possibility that RT differences also influenced the waveforms on error trials. To rule out that our results reflect a confound with between-condition differences in RT, we reanalyzed the data after matching RTs between errors and correct trials in the two speed pressure conditions. Note that although RT matching produces trial sets with similar RTs, these trial sets still differ with respect to whether participants were under low speed pressure or high speed pressure (i.e., whether participants adopted a high or low response criterion), thus leaving our experimental manipulation intact. Table 2 illustrates latencies calculated for the RT matched trials. Due to the strong RT differences, matching was not perfect. Whereas RT differences between correct trials of the two speed pressure conditions were not significant anymore, *F*_(1, 17)_ = 1.52, *p* = 0.23, a marginally significant speed pressure effect of 47 ms remained for error trials, *F*_(1, 17)_ = 4.02, *p* < 0.10. Interestingly, equalizing RTs of the primary task also abolished the effects of speed pressure effects on signaling latency, *F* < 1. This finding might indicate that signaling latencies and primary task RTs are additionally correlated due to other variables than response criterion. If RT matching eliminated differences in response criterion, this should have eliminated any Pe differences, which was not case, as we will see in the next analysis.

**Table 2:** **Behavioral performance in matched conditions**.

	**HighSP**		**LowSP**			
	**Mean**	**SE**	**Mean**	**SE**	***F*_(1, 17)_**	***p***
**PRIMARY TASK PERFORMANCE**						
RT correct. (ms)	375	33	393	41	1.52	0.23
RT error (ms)	346	29	393	42	4.02	0.06
**ERROR SIGNALING PERFORMANCE**						
Latency (ms)	676	41	702	51	0.18	0.68

Primary task response times (RTs) and error signaling latency after matching RTs for the two speed pressure conditions.

Note: SE, standard error of the mean; lowSP, low speed pressure; highSP, high speed pressure.

**Figure 4:**
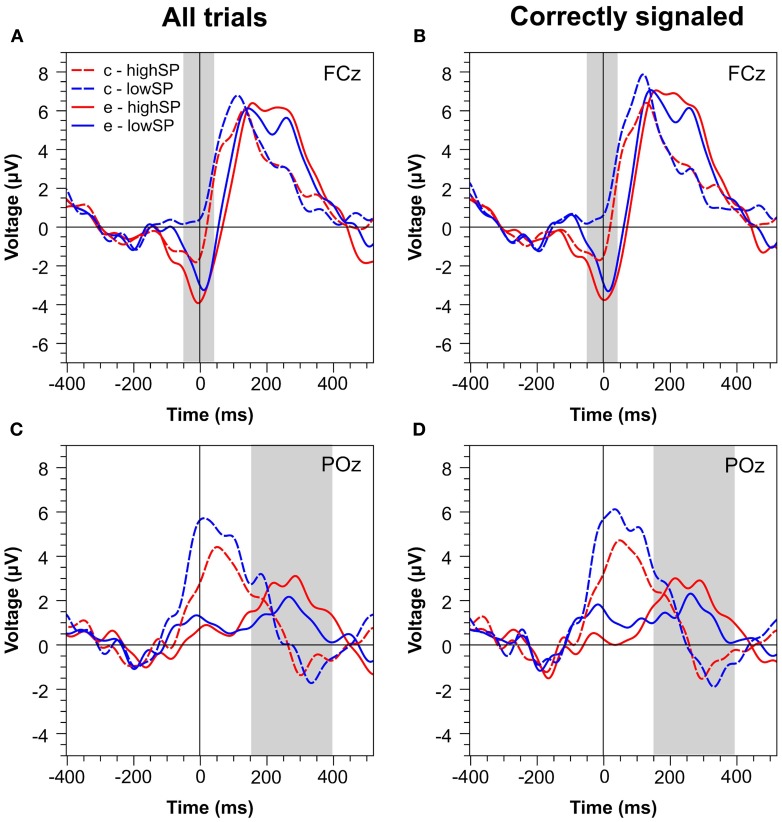
**Response-locked ERPs for RT matched data separately for the highSP and lowSP conditions**. **(A,C)** Mean ERP waveforms at electrodes FCz and POz for all errors and correct responses. **(B,D)** Mean ERP waveforms at electrodes FCz and POz for signaled errors and unsignaled correct responses. Zero indicates the time of the response. HighSP = high speed pressure. LowSP = low speed pressure.

**Figure 5:**
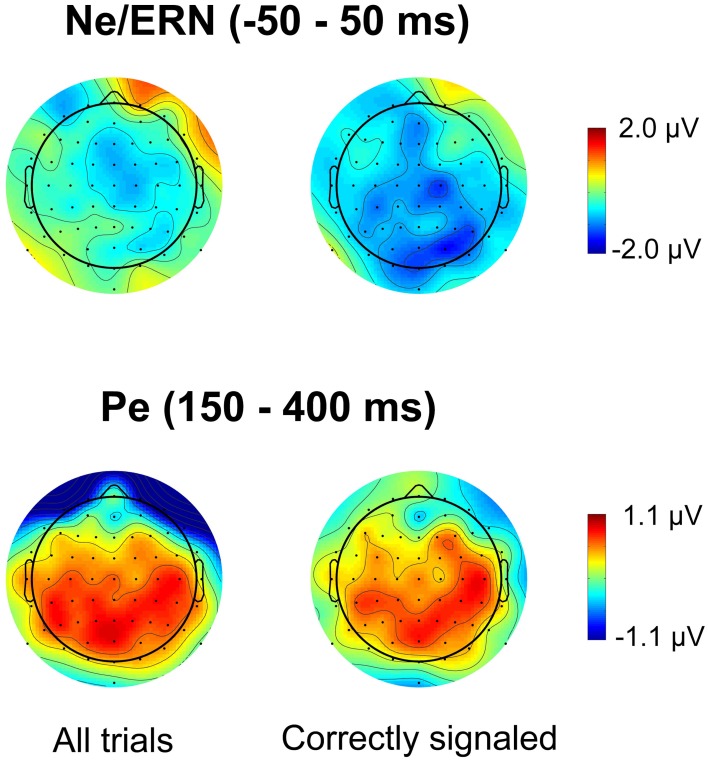
**Spatial distribution of ERPs in RT matched data for the difference between errors in the lowSP condition and errors in the highSP condition**. *Upper row:* Time period of the Ne/ERN (−50 – 50 ms). *Lower row:* Time period of the Pe (150–400 ms). *Left column:* Data from all errors. *Right column:* Data from signaled errors. HighSP = high speed pressure. LowSP = low speed pressure.

Figures 4 and 5 present waveforms and spatial distributions for the RT-matched data. Although RT matching was imperfect, effects such as the shifted ERP latencies for correct trials disappeared, suggesting that these effects were due to RT differences in the primary task. Crucially, however, amplitude differences in the Pe range of error trials between the speed pressure conditions were even slightly increased after RT matching. Again, the Pe was decreased for the lowSP condition relative to the highSP condition, and this difference was significant for all trials (1.26 μV vs. 2.21 μV), *F*_(1, 17)_ = 7.97, *p* < 0.05, as well as for correctly signaled trials (1.27 μV vs. 2.20 μV), *F*_(1, 17)_ = 5.44, *p* < 0.05, at channel POz. For the Ne/ERN, we now obtained a nonsignificant difference at channel FCz for all trials (−2.19 μV vs. −3.03 μV), *F*_(1, 17)_ = 2.03, *p* = 0.17, as well as for correctly signaled trials (−2.15 μV vs. −3.01 μV), *F*_(1, 17)_ = 1.61, *p* = 0.22.

Taken together, the analyses of response-locked ERPs suggest that less frequent and slower error signaling in the lowSP condition was associated with a reduced Pe amplitude. This reduced amplitude was obtained if all error trials were considered as well as if only correctly signaled error trials were considered, and thus seems not to reflect the decreased rate of signaled trials in the lowSP condition. In contrast, the Ne/ERN did not differ significantly between speed pressure conditions, a result which once again speaks against a direct relation between the Ne/ERN and error signaling. In the following analyses, we apply single-trial analysis to further investigate the relation between the Pe and error signaling.

### Single-trial analysis

**Figure 6:**
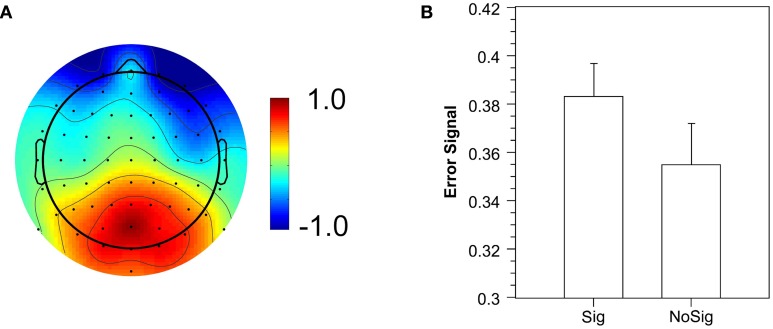
**Error signal extracted by single-trial analysis**. **(A)** Spatial distribution of the error signal as illustrated by normalized coupling coefficients. **(B)** Mean error signal for signaled (Sig) and unsignaled (NoSig) errors.

To obtain single-trial estimates of the Pe, we trained a classifier to differentiate between errors and correct trials, and used its prediction value as a single-trial measure of the error signal (Parra et al., 2002). The classifier significantly discriminated between correct and error trials (*Az* = 0.612; critical value for *p* = 0.05: 0.576; critical value for *p* = 0.01: 0.606). Figure 6A illustrates the spatial distribution of the discriminating component. It reveals a posterior distribution of weights with a peak around electrode POz, which replicates the results of Steinhauser and Yeung (2010). In a next step, we extracted the mean error signal to obtain an estimate of the single-trial Pe amplitude. In further analyses, two participants had to be excluded because they had either no signaled (*n* = 1) or no unsignaled artifact-free error trial (*n* = 1) in one of the conditions. The mean error signal for the remaining participants was significantly larger for signaled errors than for unsignaled errors (Figure 6B), *F*_(1, 15)_ = 7.62, *p* < 0.05. The logit regression analysis revealed that the error signal significantly predicted the occurrence of error signaling (beta = 4.99), *F*_(1, 15)_ = 5.94, *p* < 0.05. Both results demonstrate that the strength of the error signal predicts whether a signaling response is elicited—a crucial prediction of the evidence accumulation account—and thus replicates the findings of Steinhauser and Yeung (2010).

## Discussion

In a recent study (Steinhauser and Yeung, 2010), we proposed that error awareness—as measured by error signaling—can be described as a decision process in which evidence is accumulated until a criterion is reached. We showed that the Pe, a posterior positive ERP wave following errors, reflects the accumulated evidence that an error has occurred (rather than the outcome of such a decision). The goal of the present study was to test a specific prediction of this evidence accumulation account: that the amplitude of the Pe should vary as a function of the accumulated evidence for an error. To this end, we investigated the effects of manipulating the SAT on error signaling and the Pe. We predicted that low speed pressure in the primary task should be associated with delayed and weaker evidence for an error and, thus, with smaller Pe amplitudes. This prediction was derived from a recent modeling study (Steinhauser et al., 2008) showing that two theoretical accounts of error detection—conflict monitoring and response monitoring—predict that error detection should be impaired when speed pressure is low as compared to when speed pressure is high^6^.

To manipulate SAT without producing confounding effects of selective attention and error significance, we used a brightness discrimination task with error signaling (Steinhauser and Yeung, 2010) and instructed participants to respond within a short or a long RT limit, thus exerting high speed pressure (highSP condition) or low speed pressure (lowSP condition). Replicating findings of Steinhauser et al. (2008), the lowSP condition was associated not only with increased RTs and decreased error rates in the primary task, but also with longer signaling latencies and decreased signaling frequencies (see also Shalgi et al., 2007). Crucially, these behavioral effects were accompanied by corresponding effects in the average amplitude of the Pe. The lowSP condition exhibited a decreased Pe amplitude relative to the highSP condition. This effect was obtained irrespective of whether all error trials were analyzed or only signaled error trials, which demonstrates that this effect does not reflect the decreased rate of signaled errors in the lowSP condition. Furthermore, this effect was not reduced after matching RTs between conditions, which demonstrates that it is not due to RT differences between the speed pressure conditions. Although RT matching only reduced differences between error RTs from 90 to 47 ms rather than eliminating it, this should have reduced the Pe effect if the effect was entirely due to RT differences. In contrast, the same effect of speed pressure on Pe was obtained when RTs were matched. Taken together, these results provide support for a crucial assumption of our evidence accumulation account of error awareness. Steinhauser et al. (2008) predicted that, with low speed pressure, performance monitoring provides less evidence for an error. The present study demonstrates that this reduced evidence is reflected in reduced Pe amplitudes, suggesting a relation between the Pe and the accumulated evidence for an error.

In further analyses, we tested another prediction of the evidence accumulation account by investigating whether the Pe amplitude can be used to predict error signaling on a trial-by-trial basis. As a single-trial measure of error-related brain activity, we used the “error signal,” that is, the prediction value of a logistic regression classifier (Parra et al., 2002, 2005) that discriminated between correct and error trials. Figure 6A suggests that the classifier is associated with the typical posterior distribution of the Pe. Replicating the results by Steinhauser and Yeung (2010), the error signal extracted in the time range of the Pe was predictive of the error signaling response. The mean error signal was larger for signaled errors than for unsignaled errors, and the error signal on single trials significantly predicted whether an error would be signaled or not.

The results of the single-trial analysis replicate the findings of Steinhauser and Yeung (2010) by showing that the Pe amplitude is a valid predictor of the occurrence of signaling responses. Recently, another study extended these results by showing that the latency of the single trial Pe can also be used to predict the latency of the error signaling response (Murphy et al., 2012). Such a finding is fully in line with the idea that the Pe is related to an evidence accumulation process. In the present study, we did not focus on signaling latencies because, as in Steinhauser and Yeung (2010), we used a prompting procedure that delays error signaling in order to avoid the time range of the Pe becoming contaminated by motor activity. Signaling latencies are less informative under these conditions because this procedure eliminates variance of signaling latencies (although not fully, as indicated by the significant effect of speed pressure on mean latencies). Given that Murphy et al. (2012) used independent component analysis and that their component has a more anterior distribution than that obtained in our studies, future research will have to show whether both components really reflect the same activity.

The combined results from the present study and our previous work (Steinhauser et al., 2008; Steinhauser and Yeung, 2010), suggest that the emergence of error awareness proceeds in at least two stages. First, internal evidence for an error is provided by an implicit performance monitoring mechanism registering errors immediately after error commission. This mechanism could be based on conflict monitoring (Yeung et al., 2004) or response monitoring (Rabbitt and Vyas, 1981; Steinhauser et al., 2008), or both. Second, the output of this process either directly generates the evidence reflected in Pe amplitude, or it causes affective responses providing this evidence. The latter is suggested by studies showing that error awareness is correlated with activity related to autonomic responses (e.g., Klein et al., 2007; Wessel et al., 2011; for an overview, see Ullsperger et al., 2010). This evidence then feeds a decision which forms the basis of error awareness and which is observed in the error signaling response.

Although SAT has been a well-known empirical phenomenon for many years (Wickelgren, 1977), it is still not fully understood. Recent evidence suggests that the brain adapts to increased speed pressure by increasing baseline activity in associative areas and the pre-supplementary motor area (pre-SMA), which is computationally equivalent to a decrease in the response criterion (Forstmann et al., 2008; Bogacz et al., 2010). The present study replicates the finding that manipulating SAT of the primary task also affects the frequency and latency of error signaling (Shalgi et al., 2007; Steinhauser et al., 2008), and additionally shows that low speed pressure decreases Pe amplitude. We assumed that this effect is mediated by the effects of SAT on conflict monitoring and/or response monitoring (Steinhauser et al., 2008). Whereas conflict monitoring assumes that an error is detected by registering conflict between an incorrect response and subsequent corrective activity (Yeung et al., 2004), response monitoring assumes that an error is detected by registering that the internal correction response has exceeded the response criterion (Steinhauser et al., 2008). Despite these differences, these two accounts share the prediction that an increased response criterion in the primary task (associated with the lowSP condition) should impair the emergence of internal evidence for an error: An increased response criterion should delay the occurrence of response conflict in the conflict monitoring model, and should delay the internal correction response exceeding this criterion in the response monitoring model. Accordingly, both models can account for the finding that Pe amplitude is reduced in the lowSP condition.

In other studies investigating the effect of SAT on error processing, various alternative accounts have been proposed. Shalgi et al. (2007) explained the effects of SAT on error signaling by assuming that arousal, and thus sustained attention, is reduced under low speed pressure, and that this is the reason why error signaling is also impaired. However, these authors manipulated SAT in a go/no-go task by either exerting speed pressure (speed condition) or by instructing participants to synchronize their response to a late stimulus offset (accuracy condition), and they argued that reduced sustained attention is a direct consequence of the monotonous rhythm induced by responding to stimulus offset (Shalgi et al., 2007, p. 122). In the present paradigm, we used a more traditional SAT manipulation, such that there is no reason why sustained attention should be reduced in the lowSP condition. Reduced sustained attention should have negative effects on both speed and accuracy rather than influencing the SAT. Instead, it is possible that a change of response criterion has contributed to the results of Shalgi et al. (2007).

Several studies have investigated the effects of SAT on error-related brain activity and found the opposite results to the present study; that is, they found that low speed pressure leads to both an increased Ne/ERN and an increased Pe (e.g., Gehring et al., 1993; Arbel and Donchin, 2009). Effects like these have typically been explained by assuming that errors are generally less significant under high speed pressure (Gehring et al., 1993), or that speed pressure impairs the determination of the correct response (Falkenstein et al., 2000). However, these explanations cannot account for the absence of such a finding in the present study. The differences across studies could reflect differences in the primary task and in the method of manipulating SAT. Previous studies have mostly used a flanker task in which selective attention is necessary to respond to the target while ignoring distractors, and SAT was manipulated by emphasizing either speed or accuracy. Yeung et al. (2004) proposed that the SAT effect on the Ne/ERN in the flanker task is due to increased selective attention when accuracy is prioritized over speed (which increases response conflict after errors). This assumption can explain why we did not find such an effect in the present paradigm, in which selective attention is less relevant and instructions focus exclusively on speed rather than accuracy. This interpretation further implies that the present results reflect the pure effect of response criterion on error processing without being contaminated by effects of attention.

The absence of an SAT effect on the Ne/ERN not only contradicts previous explanations of such a finding, it also seems to violate another prediction by Steinhauser et al. (2008). Although Ne/ERN amplitudes were not directly simulated in this study, they found that the conflict monitoring model predicted a reduced level of post-error response conflict when speed pressure was reduced. Given that the conflict monitoring framework (Yeung et al., 2004) postulates a relation between post-error response conflict and the Ne/ERN amplitude, we should have obtained smaller Ne/ERN amplitudes in the lowSP condition as compared to the highSP condition. Indeed our data showed a numerically smaller Ne/ERN amplitude in the lowSP condition which reached marginal significance only when response times were not matched. This could indicate that our manipulation was simply not strong enough to reveal an SAT effect on response conflict, and thus, on the Ne/ERN amplitude. The fact that the same manipulation revealed a significant effect on the Pe could reflect that the accumulated evidence for an error reflected by the Pe is not only provided by conflict monitoring but also (and maybe even stronger) by response monitoring (Steinhauser et al., 2008). If one assumes that the Ne/ERN is more related to conflict monitoring than to response monitoring, this could also explain why single-trial amplitudes of the Pe and the Ne/ERN are only weakly correlated across trials (Steinhauser and Yeung, 2010; Hughes and Yeung, 2011).

### Conflict of interest statement

The authors declare that the research was conducted in the absence of any commercial or financial relationships that could be construed as a potential conflict of interest.

## Footnotes

^1^Steinhauser et al. (2008) discussed the alternative idea that participants simply forgot to give a signaling response on some trials (comparable to a goal neglect; De Jong et al., 1999). However, further evidence that the decision criterion associated with error awareness does not correspond to the response criterion associated with primary task comes from Steinhauser and Yeung (2010). In this study, a manipulation of the decision criterion did not lead to a significant shift of speed-accuracy trade-off in the primary task. This suggests that, if the response monitoring account is valid, one has to assume an additional decision stage that leads to error awareness.

^2^This reasoning relies on the assumption that evidence accumulation does not stop when the decision criterion is reached and, thus, that the amount of accumulated evidence can differ across two conditions with the same decision criterion, even when considering only those trials on which the criterion is exceeded.

^3^Because the onset of the signaling prompt was fully predictable, participants sometimes initiated a signaling response that preceded the prompt. 9.6% of signaling responses were too early and preceded the prompt by an average time of 72 ms. We did not exclude these trials because these responses occurred still out of the time range of the Pe and thus did not contaminate our data.

^4^The results did not change when participants with low trial numbers were excluded.

^5^This detection criterion does not necessarily reflect the decision criterion we assumed for the error signaling process but only represents a parameter of the signal detection analysis. An increased signal detection criterion could also reflect that the underlying signal is decreased for both correct and error signals.

^6^Although these predictions were derived from simulations of a flanker task, they can be generalized to any speeded choice task. The effects of response criterion on response conflict/internal correction responses should be similar for any choice task with a self-correction tendency, irrespective of whether distractor stimuli are present or not.
